# Possibility of multivariate function composed of plasma amino acid profiles as a novel screening index for non-small cell lung cancer: a case control study

**DOI:** 10.1186/1471-2407-10-690

**Published:** 2010-12-22

**Authors:** Jun Maeda, Masahiko Higashiyama, Akira Imaizumi, Tomio Nakayama, Hiroshi Yamamoto, Takashi Daimon, Minoru Yamakado, Fumio Imamura, Ken Kodama

**Affiliations:** 1 Department of Thoracic Surgery, Osaka Medical Center for Cancer and Cardiovascular Diseases, Osaka, Japan; 2 Institute for Innovation, Ajinomoto, CO., Inc., Kawasaki, Japan; 3 Department of Pulmonary Oncology, Osaka Medical Center for Cancer and Cardiovascular Diseases, Osaka, Japan; 4 HI Department, Ajinomoto, CO., Inc., Tokyo, Japan; 5 Department of Biostatistics, Hyogo College of Medicine, Nishinomiya, Japan; 6 Center for Multiphasic Health Testing & Services, Mitsui Memorial Hospital, Tokyo, Japan

## Abstract

**Background:**

The amino-acid balance in cancer patients often differs from that in healthy individuals, because of metabolic changes. This study investigated the use of plasma amino-acid profiles as a novel marker for screening non-small-cell lung cancer (NSCLC) patients.

**Methods:**

The amino-acid concentrations in venous blood samples from pre-treatment NSCLC patients (*n *= 141), and age-matched, gender-matched, and smoking status-matched controls (*n *= 423), were measured using liquid chromatography and mass spectrometry. The resultant study data set was subjected to multiple logistic regression analysis to identify amino acids related with NSCLC and construct the criteria for discriminating NSCLC patients from controls. A test data set derived from 162 patients and 3,917 controls was used to validate the stability of the constructed criteria.

**Results:**

The plasma amino-acid profiles significantly differed between the NSCLC patients and the controls. The obtained model (including alanine, valine, isoleucine, histidine, tryptophan and ornithine concentrations) performed well, with an area under the curve of the receiver-operator characteristic curve (ROC_AUC) of >0.8, and allowed NSCLC patients and controls to be discriminated regardless of disease stage or histological type.

**Conclusions:**

This study shows that plasma amino acid profiling will be a potential screening tool for NSCLC.

## Background

Recently, computer-aided systems for data mining, for example by multivariate analysis, are now readily available and have shown promising results when applied to metabolic profiling for diagnostic purposes [1,2]. Currently, several applications of metabolome analysis based on machine learning for human cancer diagnosis using peripheral blood or urine were demonstrated [3-10].

Among metabolites, the amino-acid balance in patients with various diseases often differs from that maintained in healthy individuals, as a result of metabolic changes. Amino acids are considered to be central compounds within metabolic networks. The blood serves as the medium linking the metabolic processes in the different organs of the human body. Human amino-acid metabolism in the blood has been monitored clinically for >30 years. Fischer's ratio, which is defined as the balance between branched-chain amino acids (BCAAs) and aromatic amino acids, has been used as an indicator of both the progression of liver fibrosis and the effectiveness of drug treatment [11]. Specific abnormalities in amino-acid concentrations, as assessed using multivariate analysis, have also been reported in animal models of diabetes, in human liver fibrosis and in other pathologies [12-14].

The metabolism in cancer cells is known to be significantly altered compared with that in normal cells, and these changes are also reflected in the plasma amino-acid profiles of patients with various types of cancer. For example, a significant reduction in gluconeogenic amino acids (GAAs) and a significant increase in free tryptophan have been reported in lung cancer patients [15]. Kubota et al. used plasma amino-acid profiles to discriminate between patients with breast cancer, gastrointestinal cancer, and head and neck cancers, and healthy controls [16]. Therefore, detecting metabolic changes from amino-acid profiles could potentially be useful in cancer diagnosis.

Post-genomic technologies also offer possibilities for exploiting amino-acid profiling. Recently, novel methods for analyzing amino acids have been established using high-performance liquid chromatography (HPLC)-electrospray ionization (ESI)-mass spectrometry (MS) [17-19]. This will help to make amino-acid measurements easier and reduce both the time and the cost of analysis.

Therefore, one potentially useful metabolomics tool is the "AminoIndex", which could be a simple and versatile method for monitoring various pathological conditions [12]. Here we investigated the possibility of "AminoIndex" as a novel diagnostic method for the screening of non-small-cell lung cancer (NSCLC).

## Methods

All of the patients in the study had been diagnosed histologically with NSCLC at the Osaka Medical Centre for Cancer and Cardiovascular Diseases, Japan, between January 2006 and October 2008. While hospitalized, their informed consent for inclusion was obtained. Data from the first 141 patients enrolled between January 2006 and September 2007 were used as the study data set. A further 4,340 subjects without apparent cancers, who were undergoing comprehensive medical examinations at the Mitsui Memorial Hospital, Japan, in 2008, were recruited as control subjects. Of these, 423 were age-matched, gender-matched, and smoking status-matched with the patients in the study data set group. Data from the remaining patients and control subjects were used as the test data set. Data from an additional 15 SCLC patients, who were hospitalized at the Osaka Medical Centre for Cancer and Cardiovascular Diseases, Japan, between January 2006 and October 2008, were also used. Blood samples were collected from the controls and the NSCLC patients before any medical treatment. The study was conducted in accordance with the Declaration of Helsinki, and the protocol was approved by the ethics committees of the Osaka Medical Centre for Cancer and Cardiovascular Diseases and Mitsui Memorial Hospital. All subjects gave their informed consent for inclusion before they participated in the study.

### Analytical methods

Blood samples (5 ml) were collected from forearm veins, after overnight fasting, in tubes containing ethylenediaminetetraacetic acid (EDTA; Termo, Tokyo, Japan), and were immediately placed on ice. Plasma was prepared by centrifugation at 3,000 rpm and 4°C for 15 min, and then stored at -80°C until analysis. After plasma collection, all samples were stored and processed at the Life Science Institute of Ajinomoto Co., Inc. (Kawasaki, Japan). To reduce any bias introduced prior to analysis, samples were analyzed in random order. The plasma samples were deproteinized using acetonitrile at a final concentration of 80% before measurement. The amino-acid concentrations in the plasma were measured by HPLC-ESI-MS, followed by precolumn derivatization [17-19]. The analytical methods were described in detail previously [17]. The concentrations of amino acids in the plasma were expressed as μM.

### Statistical analysis of plasma amino-acid profile

The mean amino-acid concentrations ± standard deviations (SDs) were calculated. Differences between the plasma amino-acid concentrations in NSCLC patients and controls were assessed using the Mann-Whitney U-test and receiver-operator characteristic (ROC) curve. The area under the curve (AUC) for each ROC curve (the ROC_AUC) was calculated for each amino acid.

Principal component analysis (PCA) was also used to assess differences in the plasma amino-acid profile between the controls and the NSCLC patients, with linear combinations of all of the amino acids included as explanatory variables. In PCA analysis the plasma amino-acid concentrations were transformed using the following equation:

$$z_{i,j}=\left(x_{i,j}-{\bar{X}}_{j}\right)/\sqrt{\frac{1}{n}\sum_{i=1}^{n}{\left(x_{i,j}-{\bar{X}}_{j}\right)}^{2}}$$

where *z_i,j _*was transformed concentration of of the *i*-th sample of the *j*-th amino acid, *x_i,j _*was concentration of the *i*-th sample of the *j*-th amino acid, *n *was sample size, and ${\bar{X}}_{j}$ was the average concentration of *j*-th amino acid.

### Machine learning and validation

First, an unconditional multiple logistic regression analysis with variable selection was used to construct a criterion for distinguishing NSCLC patients from controls using the study data set with the raw plasma concentrations of 21 amino acids as explanatory variables. The candidate variables of most appropriate logistic regression model, which had the minimum Akaike's information criterion (AIC) value, were selected from among all of the possible combinations in which the number of variables was below seven. A leave-one-out cross-validation (LOOCV) was performed to correct potential over-optimization for all models in parallel. Briefly, one sample was omitted from the study data set, and the logistic regression model was calculated for the remaining samples, to estimate coefficients for each amino acid. The logistic regression function values for the left-out sample were calculated based on the model. This process was repeated until every sample in the study data set had been left out once, and the function values generated were then used for AIC calculation. Finally, a case-control study was utilized for our study, and so a conditional logistic regression analysis, conditioned on the matching factors (i.e., gender, age, and smoking status), was performed in order to evaluate the association between the combination of amino acids obtained above and NSCLC. The discriminant score, which was defined as a logit of the conditional logistic regression function value, was constructed as a criterion. The degree of discriminancy of this score between NSCLC patients and controls was evaluated through the ROC curve. A distinct test data set, which had not been used in the model generation, was also used to confirm the stability of the obtained model, and to calculate the ROC_AUC values for the discriminant scores.

### Subgroup analysis

To assess the effects of cancer stage and histological type, both the study data set and the test data set was stratified according to the analysis parameters. To assess the effects of cancer stage and histological type on the discriminant scores of NSCLC patients, a subgroup analysis was performed using the ROC curve, in each data set. A two-sided P value of less than 0.05 was considered to indicate statistical significance.

### Software

All statistical analyses were performed using MATLAB (The Mathworks, Natick, MA), LogXact (Cytel, Cambridge, MA), and GraphPad Prism (GraphPad Software, La Jolla, CA).

## Results

### Characteristics of patients and control subjects

The study data set comprised 141 patients with NSCLC, and 423 age-matched, gender-matched, and smoking status-matched control subjects, whereas there were 162 patients and 3,917 controls in the test data set; a further 15 SCLC patients were also included (Table 1). Among the patients, 28% and 36% were non-smokers in the study and test data sets, respectively, whereas almost 50% of the control subjects were non-smokers (Table 1). There were no significant differences in body mass index (BMI) between the patients and the control subjects (Table 1). In both the study and test data sets ~50% of the patients were categorized as having stage I disease, ~5% as stage II, ~25% as stage III and ~20% as stage IV (Table 1). The Eastern Cooperative Oncology Group performance status (ECOG) score of most patients was 0 or 1; hence, the majority of the patients were asymptomatic or symptomatic but completely ambulatory (Table 1). The histological type was adenocarcinoma in almost 75% of the patients and squamous cell carcinoma in almost 25%, the other types present included large-cell carcinoma, adenosquamous carcinoma, pleomorphic carcinoma and mucoepidermoid carcinoma (Table 1).

**Table 1:** Characteristics of study participants

		Study data set		Test data set		SCLC
		Controls	Patients	Controls	Patients	patients
Number	Total	423	141	3917	162	15
	(Male, Female, Unknown)	(279,144)	(93,48)	(2363,1554)	(103,55,4)	(15,0)

Age, y	Mean (SD)	61.1(8.7)	62.7(9.2)	52.6(10.8)	65.7(10.4)*	66.8(8.1)*
	Range	32~82	34~83	23~88	34~83	50~76

BMI	Mean (SD)	23.0(3.1)	22.6(2.8)	22.7(3.2)	22.8(3.1)	22.7(3.5)
	Range	16.5~36.4	15.4~29.8	14.0~41.3	15.8~35.1	17.7~30.7

Smoking status	Never	126	42	2020	55	0
	Ex	45	15	1304	25	5
	Current	237	79	554	81	10
	Unknown	15	5	39	1	0

Performance	0		95		129	6
	1		41		31	5
	> 1		2		0	2
	Unknown		3		2	2

Stage**	I		69		93	6
	II		8		16	0
	III		39		30	6
	IV		25		12	3
	Unknown		0		11	0

Histology	Adenocarcinoma		100		123	
	Squamous cell carcinoma		36		33	
	Others		4		5	
	Unknown		1		1	

* Significant at *p *< 0.001 in *t-*test

** In principle, stage indicates pathological stage (p-stage). In some advanced stage (III and IV) patients who had not undergone pathological examinations, clinical stage (c-stage) is indicated instead.

### Changes in amino-acid concentrations in NSCLC patients

In the study data set, the plasma concentration of His was significantly lower, and those of Ser, Pro, Gly, Ala, Met, Ile, Leu, Tyr, Phe, Orn, and Lys were significantly higher, in NSCLC patients than in controls (Table 2).

**Table 2:** Plasma amino-acid concentration

	Plasma concentration, μM				
Amino acid	Patients (n = 141)		Controls (n = 423)		*p *value
	Mean	SD	Mean	SD	
Thr	115.6	28.6	115.9	25.4	0.92
Ser	117.1	19.9	111.4	17.9	0.003
Asn	45.1	8.2	44.8	7.2	0.72
Glu	46.7	19.4	45.7	19.5	0.60
Gln	580.5	93.3	587.9	83.5	0.40
Pro	168.0	43.6	150.9	41.4	< 0.001
Gly	263.6	63.2	237.0	57.3	0.000
Ala	422.3	97.4	383.7	88.9	0.000
Cit	34.4	10.6	33.2	8.3	0.20
ABA	24.2	8.4	23.2	6.9	0.20
Val	244.8	47.5	239.8	46.1	0.28
Met	29.4	6.1	28.0	5.2	0.013
Ile	84.3	22.1	69.7	17.5	< 0.001
Leu	131.8	34.3	122.4	27.6	0.003
Tyr	80.7	15.6	75.8	15.5	0.001
Phe	67.9	12.2	63.9	11.2	0.001
His	77.3	15.0	80.8	10.7	0.010
Trp	59.3	12.0	59.8	10.9	0.67
Orn	67.6	19.7	54.4	12.3	< 0.001
Lys	211.5	36.2	200.3	34.1	0.001
Arg	101.3	21.6	98.1	17.8	0.12

Study data set was used.

Amino acids in the human body undergo interdependent regulation; comparing single amino-acid concentrations between controls and patients might thus be insufficient to elucidate any changes in plasma amino-acid profiles associated with cancer development. Changes in the balance of the plasma amino acids in the study data set were therefore investigated using principal component analysis (PCA) in the current study. Five PCs with eigenvalues >1 were identified (Table 3). To evaluate their performance, the Mann-Whitney *U*-test was used to compare each PC score between the controls and NSCLC patients. Three of the PCs showed significant *p *values (< 0.001): PC1, PC3, and PC5 (Table 3). The contributing amino acids for the PCs that had a variance of >0.05 were then extracted; the results identified Ala, Val, Met, Ile, Leu, Tyr, Phe, Trp, and Lys as contributing factors for PC1, Cit, His, Trp, Orn, and Arg as contributing factors for PC3, and Ser, Gly, Cit, His, and Arg as contributing factors for PC5 (Table 3). As a result, fifteen amino acids (Ser, Gly, Ala, Cit, Val, Met, Ile, Leu, Tyr, Phe, His, Trp, Orn, Lys, and Arg) were identified as whose profile in plasma were associated with NSCLC (Table 3).

**Table 3:** PCA of plasma amino-acid profile of study data set

	PC1	PC2	PC3	PC4	PC5
Thr	-0.204	**0.266**	-0.187	0.096	-0.100
Ser	-0.123	**0.400**	-0.016	**-0.240**	**-0.369**
Asn	-0.211	**0.296**	-0.269	-0.040	-0.031
Glu	-0.146	**-0.374**	0.089	0.072	-0.132
Gln	-0.128	**0.290**	-0.032	0.078	0.169
Pro	-0.211	-0.064	0.153	**0.490**	-0.214
Gly	0.021	**0.384**	0.154	0.073	**-0.435**
Ala	**-0.240**	-0.057	-0.136	**0.295**	-0.220
Cit	-0.138	0.214	**0.379**	0.141	**0.394**
ABA	-0.177	-0.002	-0.033	**-0.396**	-0.212
Val	**-0.287**	-0.190	0.033	-0.235	-0.034
Met	**-0.309**	0.095	-0.101	0.052	0.060
Ile	**-0.294**	**-0.226**	0.205	-0.127	-0.118
Leu	**-0.304**	**-0.228**	0.054	**-0.258**	-0.029
Tyr	**-0.269**	-0.089	-0.005	**0.353**	-0.086
Phe	**-0.240**	-0.117	-0.066	0.005	0.163
His	-0.188	0.088	**-0.484**	-0.010	**0.256**
Trp	**-0.231**	-0.109	**-0.328**	0.090	0.112
Orn	-0.219	0.104	**0.427**	0.041	-0.053
Lys	**-0.231**	0.075	0.153	**-0.367**	0.107
Arg	-0.176	**0.225**	**0.256**	0.020	**0.421**
					
Eigenvalue	5.897	2.346	1.369	1.214	1.167
*p *value	< 0.001	0.99	< 0.001	0.23	< 0.001

PCs that had eigenvalues of >1 are indicated. Bold numbers indicate the amino acids of those PCs extracted that had a variance of >0.05. The Mann-Whitney *U*-test *p *values for comparisons of each PC score between the controls and NSCLC patients are shown.

### Classifier for discriminating NSCLC patients

**Figure 1:**
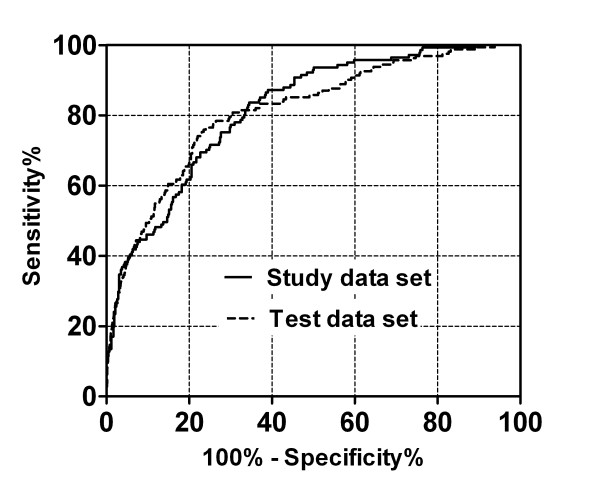
**ROC curves for discriminant scores for the discrimination of NSCLC**.

The results described so far suggested that it should be possible to improve the discrimination between cancer patients and normal controls by deriving multivariate functions, using the raw plasma amino-acid concentrations as explanatory variables, which would summarize the changes in metabolic status. Multiple logistic regression analyses by unconditional and conditional likelihood methods were therefore performed with variable selection and LOOCV cross-validation, using the study data set (as described in the Methods). The resulting conditional logistic regression model included six amino acids: Ala (*p *= 0.007), Val (*p *< 0.001), Ile (*p *< 0.001), His (*p *= 0.035), Trp (*p *= 0.027) and Orn (*p *< 0.001). The area under the curve (AUC) of the ROC for the discriminant score was 0.817 in the study data set (Figure 1).

Furthermore, to verify the robustness of the resulting model, a ROC curve was generated using the split test data set, which had not been used to construct the model. A ROC_AUC of the ROC for the discriminant score was 0.812 in the test data set (Figure 1), again demonstrating that the obtained model performed well.

### Subgroup analysis of the discriminant scores

From the point of view of cancer screening, attention might be paid to whether or not the obtained model also provides sufficient discriminating power to extract effectively patients with early-stage cancer and for all histological types. Thus, to investigate the consistency of the results based on the discriminant scores among different subpopulations defined by cancer stage and histological type, a subgroup analysis was performed using both study data set and the test data set. The discriminant scores of the SCLC patients were also calculated to verify whether the obtained model could discriminate them from the controls.

**Figure 2:**
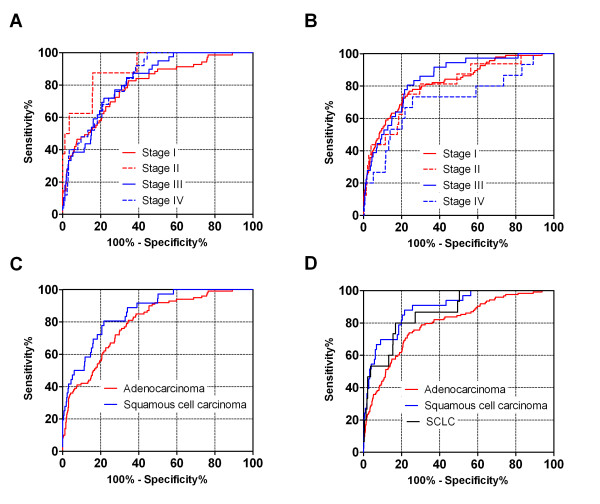
**ROC curves for discriminant scores subgrouped by cancer stage and histological type**. A. ROC curves for cancer stage of study data set. B. ROC curves for cancer stage of test data set. C. ROC curves for histological type of study data set. D. ROC curves for histological type of test data set (including SCLC patients).

Interestingly, it was suggested that the model could discriminate lung cancer patients regardless of cancer stage or histological type. Using the discriminant scores, the ROC_AUCs were 0.796 (study data set) and 0.817 (test data set) for stage I patients, 0.906 (study data set) and 0.801 (test data set) for stage II patients, 0.823 (study data set) and 0.843 (test data set) for stage III patients, and 0.836 (study data set) and 0.713 (test data set) for stages IV patients (Figure 2A, B). The model would thus be expected to be effective in detecting early, as well as advanced, cancers. We also demonstrated that the model could detect both adenocarcinomas and other histological types of cancer equally well: the ROC_AUCs were 0.795 (study data set) and 0.796 (test data set) for adenocarcinoma, and 0.860 (study data set) and 0.892 (test data set) for squamous cell carcinoma (Figure 2C, D). Furthermore, the distribution of the discriminant scores for SCLC patients was similar to that for NSCLC patients, with a ROC_AUC of 0.877 (Figure 2D).

## Discussion

Lung cancer has been the leading cause of cancer death since 1998 and >60,000 patients have died since 2005 in Japan. The 5-year survival rate for patients undergoing surgery is only 61%, and an accurate screening method for lung cancer would be an important advance [20]. In Japan, chest X-rays and sputum cytology are used for screening lung cancer. Although chest X-rays are useful for detecting peripheral lung cancer, two-thirds of patients diagnosed in this way have associated metastases, and this method is not sufficient to detect the early stages of the disease [21]. In addition, highly skilled staffs are required to achieve sufficient accuracy. Sputum cytology might be useful for detecting upper respiratory-tract carcinoma, but this method has been reported to be inadequate for detecting peripheral lung cancer and lung cancer in asymptomatic non-smokers [21]. Recently, low-dose helical computed tomography (CT) was reported to be capable of detecting small, early lung cancers in high-risk populations; however, it is not known whether using this method would affect the mortality rate due to lung cancer or whether it would be cost-effective [22].

In comparison to those methods, the "AminoIndex" would be easier to use, as it involves a relatively simple plasma assay, imposes a lower physical burden on patients and does not require advanced technical skills to perform [12]. The current study demonstrated that plasma amino acid profiles were associated with NSCLC. The ROC_AUCs were 0.817 for the study data set under the conditional logistic regression analysis conditioned on the matching factors (Figure 1). Okamoto et al. recently reported that plasma amino-acid profiles might be used to screen colorectal and breast cancer [23]. Despite the smaller sample size, they reported ROC_AUCs of 0.860 (with study data) and 0.910 (with test data) for colorectal cancer patients, and 0.906 (with study data) and 0.865 (with test data) for breast cancer patients [23]. Our current study achieved similar discrimination power using data set with a larger sample size under controlling for potential confounders, thereby demonstrating the robustness of the model.

Many reports have shown that the metabolism, including that of amino acids, is notably altered in cancer cells [4,24-26], and that the plasma amino-acid profiles are also changed [15,16,27-30]. Cascino et al. described significant increases in levels of Trp, Glu and Orn in lung cancer patients [15]. Proenza and colleagues also reported an increased level of Orn in patients with lung cancer [29]. Naini et al. reported reduced levels of plasma Arg in lung cancer patients [31].

Changes in the amino-acid balance and an increase in gluconeogenesis have been well documented, especially in cachexic patients with advanced cancer [32,33]. In the current study, the obtained model identified patients at all stages of lung cancer and without cachexia equally well, suggesting that the method did not rely on detecting metabolic abnormalities associated with malnutrition, which might be present in advanced cancer patients (Figure 2A, B). Hirayama et al. reported no significant correlation between the levels of metabolites, including several amino acids, and the patients' tumour stage [24]. And it was also reported that amino acids were frequently identified compounds among whole metabolites in blood in relation to cancer [3,8]. The metabolism of specific amino acids is known to be associated with specific organs, such as muscle, liver or kidney, changes in the levels of amino acids are affected by their metabolism in, and excretion from, multiple organs of the body. Although it remains unclear how the metabolic changes occurring in tumour cells affect the systemic, plasma amino-acid profile, these results show that the metabolic changes caused by cancer development are at least partially responsible for the changes in plasma amino-acid profile seen even in lung cancer patients with early stage cancer. So, profiling the plasma free amino acids is similar to monitoring metabolic networks in multiple organs and it might better allow us to detect particular conditions in specific organs.

Since this study was designed as a case-control study, the obtained model could not be directly applied to further observation or prediction even though the robustness of the model was preliminarily demonstrated. Therefore model construction and validation using cohort with larger samples will be necessary to clarify its utility. Nonetheless, we believe that this screening technique could be a straightforward diagnostic method for the management of lung cancer.

## Conclusions

The current study demonstrated that the plasma amino-acid profile of NSCLC patients differed from that of healthy subjects. And we showed that the multivariate classifier might be effective for discriminating lung cancer patients. Although further prospective validation will be necessary in the future, this method might be an effective and convenient screening tool for lung cancer patients.

## Competing interests

We declare that we are participants in the "AminoIndex" research consortium organized by Ajinomoto, and that we have all seen and approved the final version of this manuscript. Akira Imaizumi and Hiroshi Yamamoto are employees of Ajinomoto. Masahiko Higashiyama, Fumio Imamura and Akira Imaizumi have applied for patents for plasma amino-acid profiling using multivariate analysis as a diagnostic procedure.

## Authors' contributions

AI and HY designed this case control study. JM, MH, TN, MY, FI and KK coordinated the study and collected the background data on the subjects. HY also coordinated the study, and supervised the collection of control data. JM, TD, and AI provided data analysis and wrote the manuscript. JM, MH, AI, TN, HY, TD, MY, FI and KK provided final reviews and approval of the manuscript. All authors read and approved the final paper.

## Pre-publication history

The pre-publication history for this paper can be accessed here:

http://www.biomedcentral.com/1471-2407/10/690/prepub
